# Molecular characterization of host-parasite cell signalling in *Schistosoma mansoni* during early development

**DOI:** 10.1038/srep35614

**Published:** 2016-10-20

**Authors:** Margarida Ressurreição, Firat Elbeyioglu, Ruth S. Kirk, David Rollinson, Aidan M. Emery, Nigel M. Page, Anthony J. Walker

**Affiliations:** 1 Molecular Parasitology Laboratory, School of Life Sciences, Pharmacy and Chemistry, Kingston University, KT1 2EE, United Kingdom; 2 Department of Life Sciences, The Natural History Museum, London, SW7 5BD, United Kingdom

## Abstract

During infection of their human definitive host, schistosomes transform rapidly from free-swimming infective cercariae in freshwater to endoparasitic schistosomules. The ‘somules’ next migrate within the skin to access the vasculature and are surrounded by host molecules that might activate intracellular pathways that influence somule survival, development and/or behaviour. However, such ‘transactivation’ by host factors in schistosomes is not well defined. In the present study, we have characterized and functionally localized the dynamics of protein kinase C (PKC) and extracellular signal-regulated kinase (ERK) activation during early somule development *in vitro* and demonstrate activation of these protein kinases by human epidermal growth factor, insulin, and insulin-like growth factor I, particularly at the parasite surface. Further, we provide evidence that support the existence of specialized signalling domains called lipid rafts in schistosomes and propose that correct signalling to ERK requires proper raft organization. Finally, we show that modulation of PKC and ERK activities in somules affects motility and reduces somule survival. Thus, PKC and ERK are important mediators of host-ligand regulated transactivation events in schistosomes, and represent potential targets for anti-schistosome therapy aimed at reducing parasite survival in the human host.

Schistosomes are formidable multicellular blood parasites. Human-infective species such as *Schistosoma mansoni* and *S. haematobium* penetrate the skin as cercariae in freshwater, transform into skin schistosomules (somules) and then migrate in the vasculature, and develop further into male and female immature worms. These worms pair, mature and migrate to the egg laying site; a female can produce hundreds of eggs each day many of which are released in the faeces or urine enabling parasite transmission *via* a snail intermediate host^1^. Eggs not expelled from the definitive host get trapped in tissues and elicit chronic immune responses causing granulomas that result in the neglected tropical disease (NTD) human schistosomiasis^2^. The importance of this NTD is considerable; approximately 230 million people are infected across 76 developing countries and 0.8 billion are at risk of infection^3^.

Upon skin invasion the schistosome must quickly adapt to survive in the new environment of the human host. To facilitate survival, immunomodulatory molecules are released by cercariae during invasion^4^ and each cercaria undergoes complex transformation into a biochemically distinct skin somule^5^ which develops a specialised syncytial tegument that remains into adulthood. This unique host-interactive layer has been the focus of much research, especially because it expresses potential drug and vaccine targets^6^,^7^,^8^,^9^,^10^. Even at this early stage of parasitism, the schistosome likely exploits host-signalling molecules to support its development and sustain homeostasis, and host-parasite communication could occur *via* the tegument. However, the extent to which human host molecules influence schistosome cellular mechanisms remain largely unknown.

In eukaryotes, protein kinase C (PKC) and extracellular signal-regulated kinase/mitogen-activated protein kinase (ERK/MAPK) regulate diverse processes such as growth, development and differentiation, the cell cycle, motility, apoptosis and survival^11^,^12^. These intracellular signalling proteins/pathways are activated by stimuli including ligands that bind to transmembrane G-protein coupled receptors (GPCRs) and receptor tyrosine kinases (RTKs). In *S. mansoni*, four PKC-like proteins have been identified which share homology to human PKCs particularly within their functional domains: two conventional PKCβ, one novel PKCε, and one atypical PKCζ^13^,^14^,^15^, with PKCε also been designated PKCη^16^. Putative upstream PKC regulators such as phospholipase C (PLC) also exist^17^. In respect of the ERK pathway, which comprises Ras as a monomeric GTP-ase switch protein, Raf as a MAPKKK, MAPK/ERK kinase (MEK) as a MAPKK, and ERK as a MAPK, in *S. mansoni* a Ras GTPase activator protein has been detected^18^, and Ras and ERK homologues characterized^15^,^18^,^19^.

Signalling across cellular plasma membranes is considered to occur through dynamic membrane/lipid rafts, which are nanoscale microdomains present in the lipid bilayer that assemble cholesterol and sphingolipids and subsets of transmembrane or glycosylphosphatidylinisotol (GPI)-anchored proteins^20^. These structures are presumed to also selectively concentrate intracellular signalling molecules providing platforms for where protein kinases, scaffolding molecules, and substrates are brought into close proximity enabling rapid signal transduction^21^. These rafts also play a part in membrane trafficking^22^,^23^. Raft formation is most likely controlled by proteins, including caveolins and flotillins^24^ that probably organize rafts into microdomains^23^. In schistosomes putative caveolae-like structures, possibly formed by caveolin-like molecules, have been described in the surface (tegumental) membrane, and membrane fractions characteristic of detergent-insoluble glycosphingolipid-enriched membrane domains (DIGs) or detergent-resistant membranes (DRMs) have been prepared^25^, indicating lipid raft presence *in vivo*^23^.

Here we have characterized ERK and PKC signalling in somules of *S. mansoni* during early development *in vitro* and show that these pathways are transactivated by host epidermal growth factor (EGF), insulin, and insulin-like growth factor I (IGF-I). We provide evidence to further support the presence of lipid rafts at the schistosome surface and propose that signalling to kinases such as ERK occurs through these rafts. Finally we show that modulation of PKC and ERK activities affects somule motility, phenotype, and reduces somule survival.

## Results

### Characterization of PKC and ERK activation during early somule development

**Figure 1:**
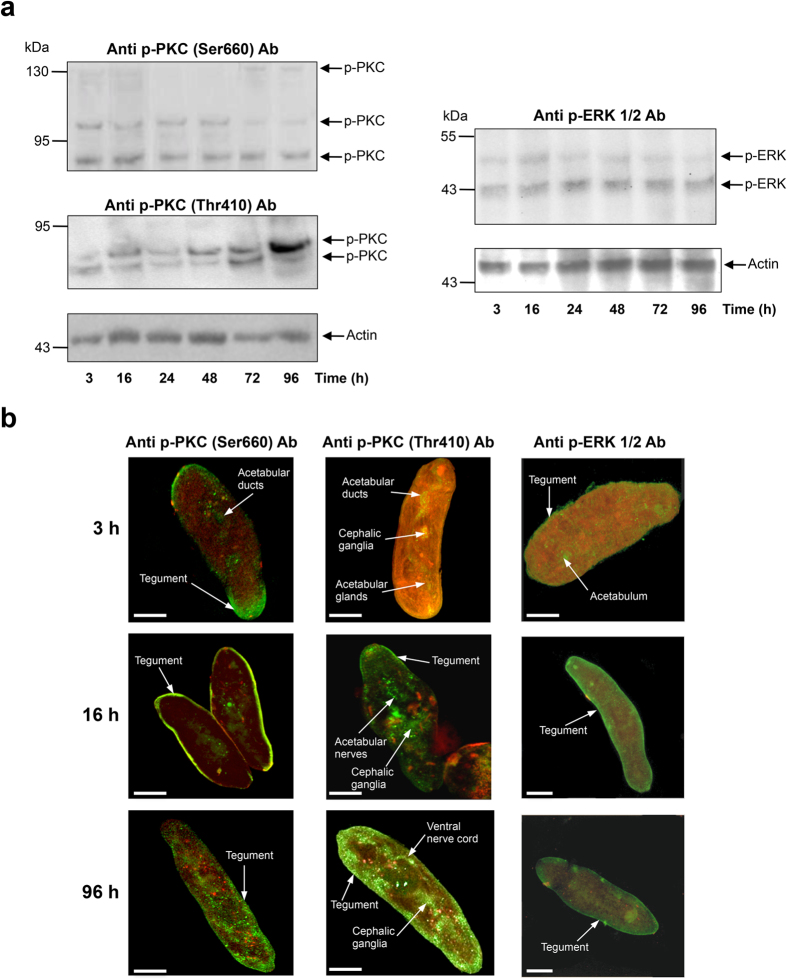
PKC and ERK activation during *S. mansoni* early somule development *in vitro*. (**a**) Detection of phosphorylated (activated) PKCs (p-PKC) and ERKs (p-ERK) in somules during 96 h culture by western blotting using anti-phospho PKC (Ser660)/(Thr410) or anti-phospho p44/42MAPK (Thr202/Tyr204) (ERK1/2) antibodies. Anti-actin antibodies were used to monitor differences in total protein loading between lanes. (**b**) *In situ* localization of activated kinases (green) within somules at 3, 16 and 96 h using anti-phospho PKC (Ser660)/(Thr410) or anti-phospho p44/42MAPK (Thr202/Tyr204) (ERK1/2) primary and AlexaFluor 488 secondary antibodies and confocal microscopy. F-actin is stained with rhodamine phalloidin (red). Representative micrographs of somules are shown as z-axis maximum projections. Bar = 25 μm.

Anti-phospho antibodies, validated by us for detecting *S. mansoni* PKCs and ERKs when in an exclusively phosphorylated (activated) state^13^,^15^, were employed here to characterize and localize the phosphorylation (activation) dynamics of PKCs and ERKs in mechanically transformed somules maintained *in vitro* for four days. Such somules are similar to skin-transformed somules and the transformation of the tegument occurs rapidly *in vitro*^26^,^27^. In immunohistochemistry, control somules probed only with secondary antibodies consistently displayed negligible fluorescence (Supplementary Figure 1). Using anti-phospho PKC (Ser660) and anti-phospho PKC (Thr410) antibodies that recognize activation motifs in *S. mansoni* PKCs that are conserved with human PKCs^15^, three phosphorylated (activated) PKCs were consistently detected in somules with apparent molecular weights of ~78, ~81, and ~116 kDa (Fig. 1a). Based on immunoreactivity profiles and conservation of amino acids within the key activation motifs these PKCs have been tentatively assigned Smp_128480 (conventional PKCβ-type), Smp_096310 (atypical PKCι-type), and Smp_176360 (conventional PKCβ-type), respectively as previously reported^15^. Each of these PKC genes are expressed in 3 h and 24 h somules (data available at GeneDB). A larger (~132 kDa) PKC-like protein was also sometimes detected, albeit weakly (Fig. 1a). Digital analysis of western blot bands from four separate experiments revealed that activation of the ~78, ~116, and ~132 kDa PKCs did not change significantly over four days when compared to 3 h somules; however, ~81 kDa PKC activation was consistently upregulated at 72 h and was sustained at 96 h when compared to 3 h somules (Fig. 1a) (~2.4 fold increase; p ≤ 0.001). Localization of activated PKCs within intact somules during early development with anti-phospho PKC (Ser660) antibodies using confocal laser scanning microscopy revealed that activated PKC was differentially distributed over time, with activation at the tegument at 3 h and 16 h, which declined thereafter, with greater activation seen within the somule body at 96 h (Fig. 1b). Using anti-phospho PKC (Thr410) antibodies, activated PKC was seen at the tegument and various internal structures throughout development with considerable sub-tegumental activation seen at 96 h, together with activation at structures resembling the nerve cords and cephalic ganglia (Fig. 1b). Similar to three of the PKCs, the activities of the ~43 kDa and ~48 kDa ERK proteins detected using anti-phospho p44/42MAPK (Thr202/Tyr204) (ERK1/2) antibodies did not change significantly during early somule development *in vitro* (Fig. 1a). Confocal microscopy analysis of somules over 96 h revealed activated ERK was generally associated with the tegument region (Fig. 1b).

### Induction of somule PKC and ERK signalling by human EGF, Insulin and, IGF-I

**Figure 2:**
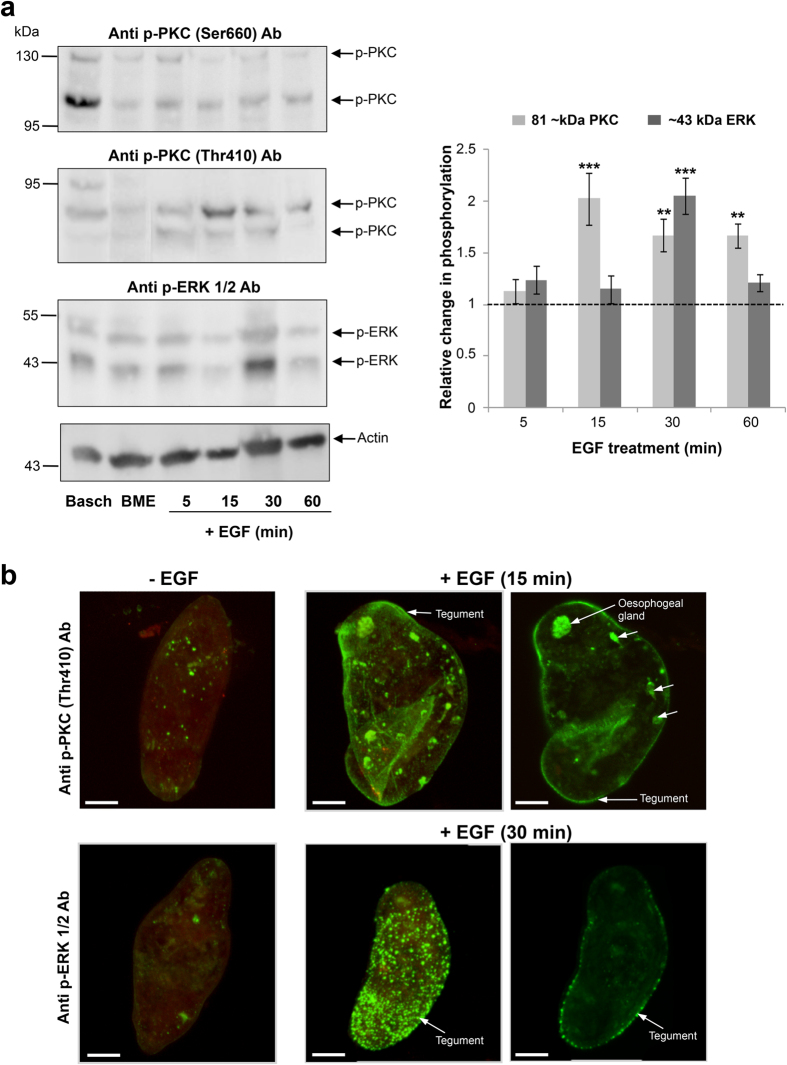
Human EGF stimulates PKC and ERK activation in *S. mansoni* somules. (**a**) 72 h *in vitro*-cultured somules, starved overnight, were exposed to EGF (15 ng/ml; 5–60 min) and phosphorylated (activated) PKCs (p-PKC) and ERKs (p-ERK) detected by western blotting with anti-phospho PKC (Ser660) or (Thr410) and anti-phospho p44/42MAPK (Thr202/Tyr204) (ERK1/2) antibodies. Control somules (in BME) remained untreated, and somules cultured in complete Basch media were also processed. Band intensities on blots were quantified and mean relative change in phosphorylation (n = 5, ±SEM; graph), normalised for actin levels, was calculated with respect to BME controls that were assigned a value of 1; **p ≤ 0.01, ***p ≤ 0.001. (**b**) *In situ* localization of activated PKC and ERK (green) within 72 h somules with or without EGF treatment using anti-phospho PKC (Thr410), anti-phospho p44/42MAPK (Thr202/Tyr204) (ERK1/2) and AlexaFluor 488 antibodies and confocal microscopy. Representative micrographs of somules are shown as z-axis maximum projections, except for the right-hand side images, which are single z-sections through the parasite. Bar = 25 μm.

We next sought to investigate the effects of host molecules on PKC and ERK signalling in somules. Three-day old somules that had been starved overnight in BME were treated with human EGF (15 ng/ml). EGF exposure resulted in a transient, significant induction of PKC (81 kDa) and ERK (43 kDa) activation, with maximal ~2-fold (p ≤ 0.001) and ~1.6-fold (p ≤ 0.001) increases seen at 15 and 30 min, respectively (Fig. 2a); activation of the other PKC and ERK proteins remained unaffected (Fig. 2a). Anti-phospho PKC (Thr410) and anti–phospho p44/42MAPK (Thr202/Tyr204) (ERK1/2) antibodies were therefore used to localize activated PKCs and ERKs in somules at these time points. EGF treatment caused a visible increase in PKC activation at the tegument and also at internal structures including a region resembling the oesophagus/oesophageal gland, particularly noticeable with deep scanning (Fig. 2b). There was also striking activation of ERK at the tegument, with a punctate distribution over the entire surface of the somule. Interestingly, enhanced activation of the 116 kDa PKC was evident when somules remained in Basch’s growth medium when compared to Eagles Basal Medium (BME) controls (Fig. 2a).

**Figure 3:**
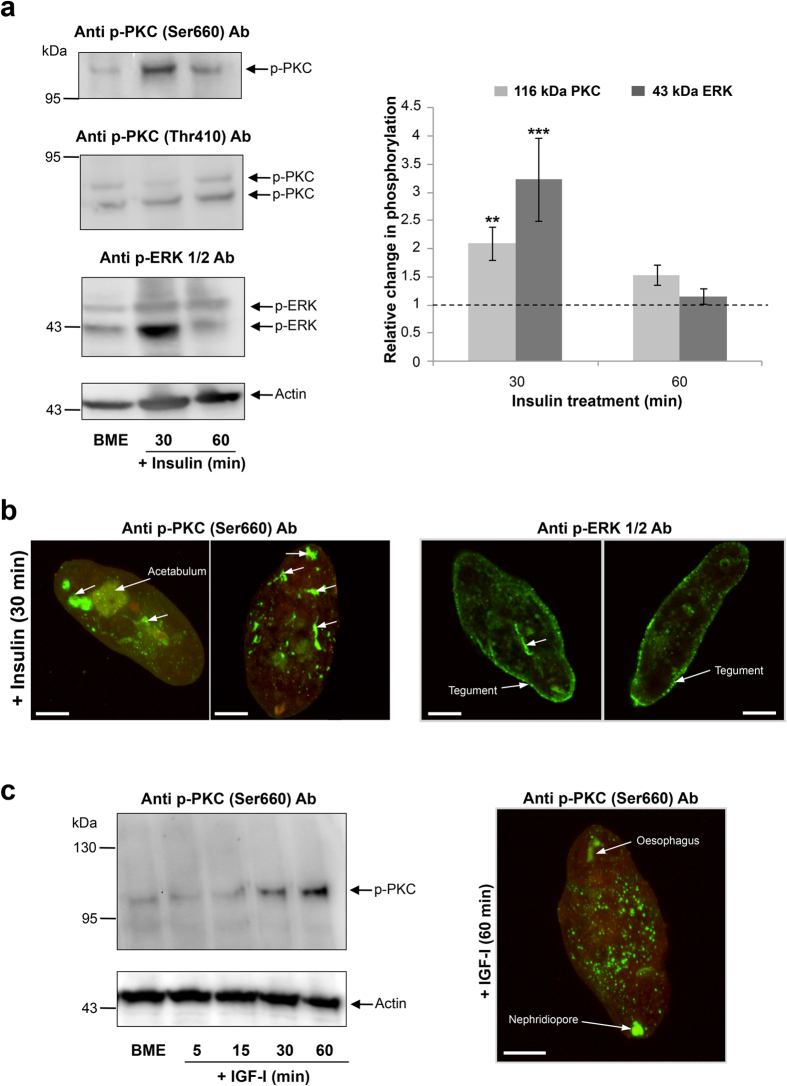
Human insulin and IGF-I stimulate PKC and ERK activation in *S. mansoni* somules. (**a**) 72 h *in vitro*-cultured somules, starved overnight, were exposed to insulin (1 μM; 30, 60 min) and phosphorylated (activated) PKCs (p-PKC) and ERKs (p-ERK) detected by western blotting with anti-phospho PKC (Ser660) or (Thr410) and anti-phospho p44/42MAPK (Thr202/Tyr204) (ERK1/2) antibodies. Control somules (in BME) remained untreated. Band intensities were quantified and mean relative change in phosphorylation (n = 6, ± SEM; graph), normalised for actin levels, was calculated with respect to BME controls that were assigned a value of 1; **p ≤ 0.01, ***p ≤ 0.001. (**b**) *In situ* localization of activated PKC and ERK (green) within 72 h somules following insulin treatment using anti-phospho PKC (Ser660), anti-phospho p44/42MAPK (Thr202/Tyr204) (ERK1/2) and AlexaFluor 488 antibodies and confocal microscopy. (**c**) Detection of activated PKC in response to IGF-I exposure with time using anti-phospho PKC (Ser660) antibodies. Representative micrographs of somules are shown as z-axis maximum projections. Bar = 25 μm.

Addition of human insulin (1 μM) to starved somules significantly increased the activation of both the ~116 kDa PKC and ~43 kDa ERK at 30 min, by ~2-fold and ~3.2-fold, respectively, declining at 60 min (Fig. 3a). The activation status of the remaining PKCs and ERK were unaffected by insulin (Fig. 3a). Moreover, immunohistochemistry with anti-phospho PKC (Ser660) antibodies (that detect the 116 kDa PKC) revealed that after 30 min, activated PKC associated with acetabulum and distinct unidentified internal structures, whereas activated ERK also localized to the tegument with punctate distribution (Fig. 3b) similar to that with EGF (Fig. 2b).

In contrast to insulin, incubation of serum-starved somules with IGF-I (15 ng/ml) resulted in the activation of only the ~116 kDa PKC at 30 and 60 min (Fig. 3c). Deep scanning revealed activation present in the parenchyma and in regions resembling the oesophagus and, possibly the nephridiopore (Fig. 3c).

### Lipid raft markers and DRM behaviour support a role for lipid raft-mediated cell signalling in *S. mansoni*

**Figure 4:**
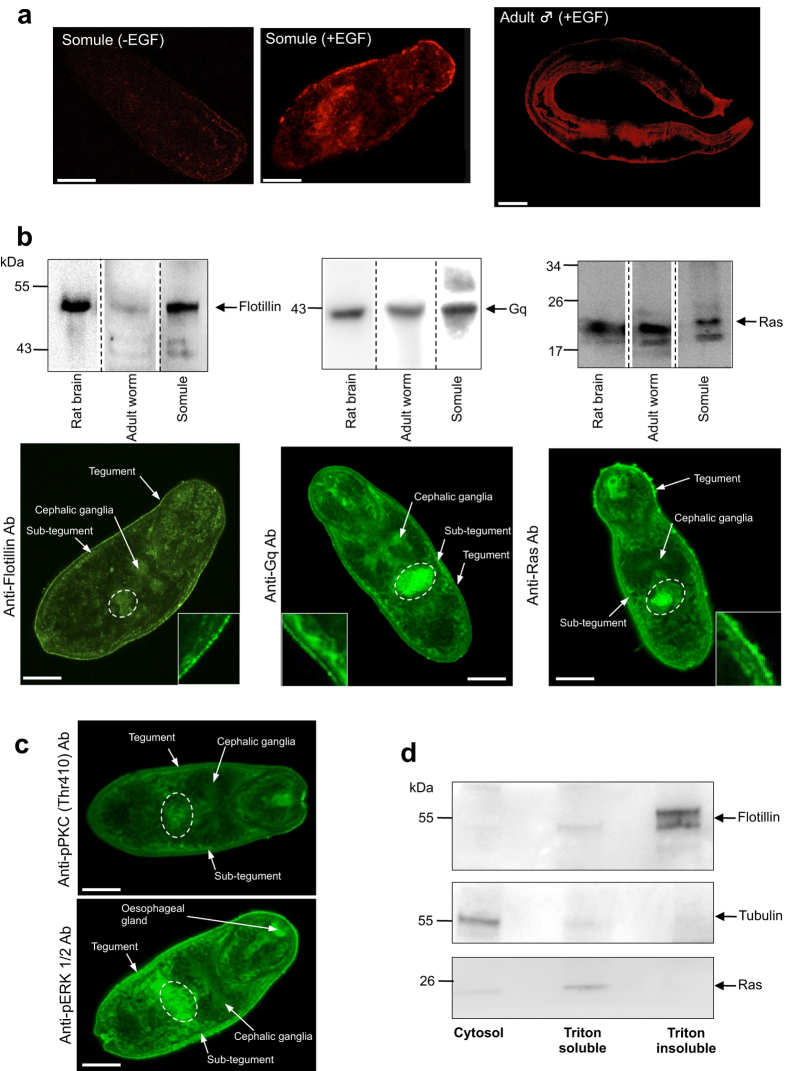
Identification of lipid rafts in *S. mansoni.* (**a**) *In situ* detection of lipid rafts in control (-EGF) 24 h somules or 24 h somules/adult male worms treated with EGF (15 ng/ml; +EGF) for 5 min, stained live with the AlexaFluor 594 lipid raft labelling kit and imaged by confocal microscopy. (**b**) Identification of flotillin, Gq and Ras in adult worms (1 pair) and 24 h somules (~1000) by western blotting (with rat brain positive control) and *in situ* localization within somules by confocal microscopy using anti-flotillin, anti-Gq, and anti-Ras antibodies. The inset panels display localization of these proteins at the tegument region. (**c**) Detailed *in situ* analysis of PKC and ERK activation in 24 h somules exposed to EGF (15 ng/ml) using anti-phospho PKC (Thr410) and anti-phospho p44/42MAPK (Thr202/Tyr204) (ERK1/2) antibodies. (**d**) Separation of triton-insoluble (detergent-resistant) membranes from triton-soluble membranes and cytosolic fraction and identification of flotillin, Ras and tubulin within each fraction by western blotting; each lane contains equal amounts of total protein (39 μg) derived from fractionation of adult worm extracts. Representative micrographs of somules are single z scans. Bar = 25 μm.

Given that one day-old somules displayed activated PKC and ERK (Fig. 1), which localized to the tegument, further experiments investigating lipid rafts were conducted on ~24 h somules that had not been cultured in serum. Similar to 3-day somules, preliminary experiments revealed that these somules also responded to EGF (data not shown). Lipid raft microdomains are characterized by their insoluble nature in non-ionic detergents as well as the presence of the constituent pentasaccharide ganglioside_GM1_. In an initial step to identify lipid rafts, somules were treated with EGF and stained with the Vybrant lipid raft labelling kit, which incorporates fluorescent cholera toxin subunit B (CT-B) that binds ganglioside_GM1_. GM1 clusters, indicative of lipid rafts, were observed at the somule surface following EGF treatment (Fig. 4a) with less staining seen without EGF. No fluorescence was observed in negative controls not incubated in CT-B (data not shown). Moreover, GM1 clusters were observed at the surface of adult *S. mansoni* (Fig. 4a).

Next, to identify possible raft marker proteins in schistosomes, protein sequences for human flotillin-1, Gq and Ras were BLASTed against the *S. mansoni* genome, alignments generated, and antibodies selected to recognize the *S. mansoni* homologues based on target sequence. *S. mansoni* flotillin-1 (Smp_016200.3) is 62% identical to human flotillin-1 (NP_005794.1; 48 kDa). Antibodies raised against amino acids 312–428 (that incorporate the conserved flotillin domain) of human flotillin-1 detected a band of ~48 kDa in somule and adult worm extracts, similar to the expected molecular weight of the *S. mansoni* protein (47 kDa). Two other flotillin splice variants, Smp_016200.1 and Smp_016200.4 of 41 kDa and 43 kDa, respectively, are predicted in the *S. mansoni* genome and might be responsible for the two lower molecular weight bands detected (Fig. 4b). *S. mansoni* G protein (Smp_005790; ~42 kDa) is 81% identical to human G protein subunit α-11 (Gq class; NP_002058) and anti-Gq/11α antibodies targeted to a region (QLNLKEYNLV) that is 100% conserved between the species recognized the putative *S. mansoni* 42 kDa protein (Fig. 4b). Finally, *S. mansoni* Ras (Smp_179910; 16 kDa), 81% similar to human K-Ras (isoform a; NP_203524), was detected at ~20 kDa by an antibody targeted to the N-terminal region of human K-, H- and N-Ras (which is 100% similar between the species) (Fig. 4b). Immunohistochemistry localized flotillin, Gq, and Ras to the somule tegument and sub-tegument regions, cells within the parenchyma tissues and also internal structures that included the cephalic ganglia and a cluster of cells proximal to the acetabulum (Fig. 4b). High-resolution imaging of the tegument region revealed punctate staining, particularly for flotillin and Gq (Fig. 4b). Detailed *in situ* analysis of activated ERK and PKC in these somules following EGF treatment revealed overall similar distribution patterns, with activated ERK also detected at the oesophageal gland region (Fig. 4c). Because enrichment of a protein in a DRM preparation shows that it is raftophillic and that it is likely to associate with lipid rafts when they form^23^ we prepared DRMs^28^ from schistosomes using triton X-100. Adult worms were used rather than somules to ensure that sufficient material was available for DRM preparation. Western blotting revealed that flotillin was enriched in the triton-insoluble DRM fraction, indicating the presence of lipid rafts, whereas Ras was found predominantly in the triton-soluble fraction and β-tubulin was exclusively in the cytosolic fraction (Fig. 4d).

**Figure 5:**
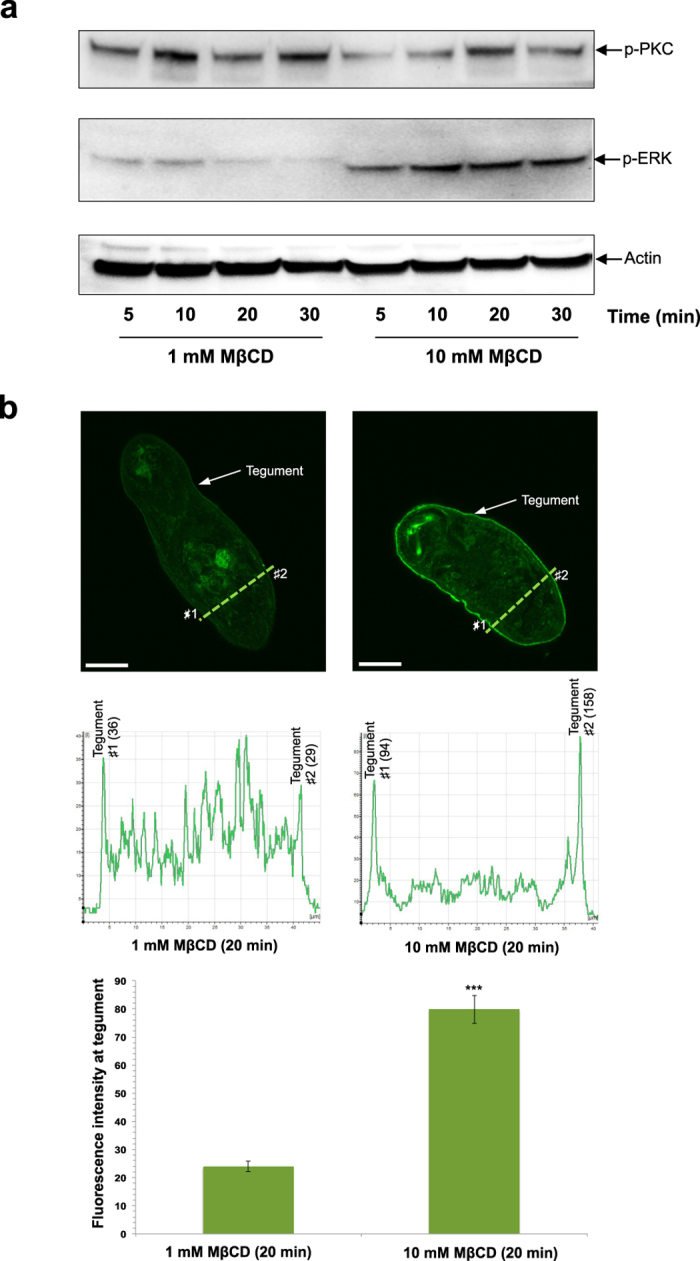
Lipid raft disruption through cholesterol depletion affects ERK signalling in *S. mansoni* somules. Somules (~1000) were incubated in 1 or 10 mM methyl-β-cyclodextrin (MβCD) for 5–30 min and subsequently exposed to 15 ng/ml EGF. Somule protein extracts were processed for western blotting with anti-phospho PKC (Thr410) or p44/42MAPK (Thr202/Tyr204) (ERK1/2) antibodies; anti-actin antibodies were used to monitor total protein loading differences between lanes. Results are representative of those seen in two independent experiments. (**b**) *In situ* analysis of ERK activation in somules exposed to 1 or 10 mM MβCD for 20 min followed by 15 ng/ml EGF, using anti-phospho p44/42MAPK (Thr202/Tyr204) (ERK1/2) antibodies. The fluorescence intensity at the tegument (e.g. at locations ♯1 and ♯2 of a single confocal z scan) was quantified (line graph) at two separate random locations for each somule (n = 15) and mean fluorescence intensity calculated ( ± SEM; bar chart). Bar = 25 μm.

Lipid rafts can be disrupted using the cholesterol depleting agent methyl-β-cyclodextrin (MβCD). We therefore next investigated whether or not raft disruption affected PKC and/or ERK signalling. Somules were treated with either high (10 mM) or low (1 mM)^29^,^30^ concentrations of MβCD for increasing durations prior to EGF exposure. ERK activation was visibly suppressed after 20 min treatment with 1 mM MβCD, when compared with earlier time points (Fig. 5a). In contrast, ERK was activated at all time points using 10 mM MβCD, when compared with 1 mM MβCD treatment (Fig. 5a). In replicate experiments, there was no consistent pattern of PKC activation/inhibition in response to treatment with MβCD (Fig. 5a). Immunohistochemistry of somules treated with 1 mM or 10 mM MβCD for 20 min prior to EGF stimulation revealed that 10 mM MβCD significantly increased ERK activation at the tegument of the parasite (Fig. 5b). These findings are consistent with lipid rafts playing a role in ERK pathway activation at the surface of somules.

### Modulation of PKC and ERK signalling in somules affects somule morphology and motility and reduces their survival

**Figure 6:**
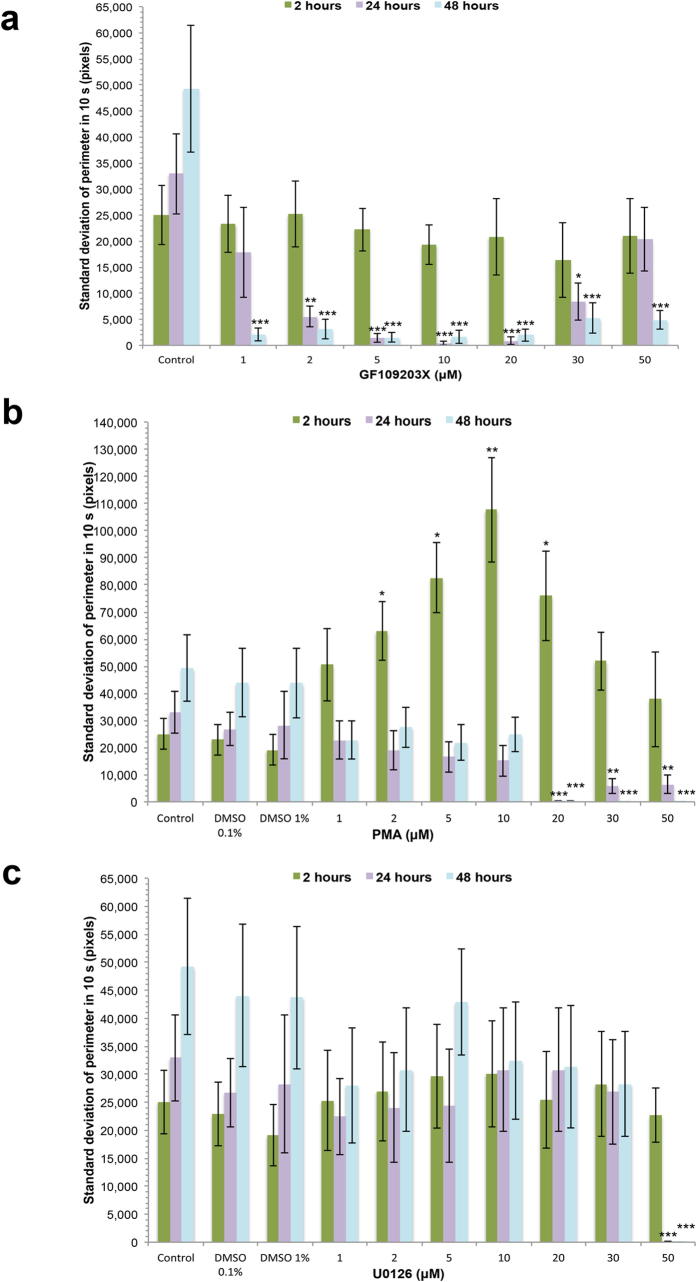
Modulation of PKC or ERK signalling affects the motility of *S. mansoni* somules. Somules were incubated in increasing concentrations of (**a**) GF109203X, (**b**) PMA, or (**c**) U0126 and movies captured at 2 h, 24 h, or 48 h. Motility (standard deviation of perimeter) of somules was determined using ImageJ over 10 s at each time point, with mean values (±SEM; n = 30) determined from three independent experiments. *p≤0.05, **p≤ 0.01, and ***p≤ 0.001, when compared to respective control values as follows: GF109203X = media (control); PMA and U0126: 1–30 μM = 0.1% DMSO, 50 μM = 1% DMSO.

We have previously validated GF109203X and PMA, and U0126, for use in *S. mansoni* as modulators of PKC and ERK activation, respectively^13^,^15^. In live parasites, GF109203X and U0126 attenuate *S. mansoni* PKC and ERK activation, respectively, and PMA activates PKC/ERK, but down-regulates PKC after prolonged (overnight) exposure^13^,^15^. Given that human EGF, insulin and IGF-I activated these pathways in somules, we aimed to understand the importance of these pathways to somule phenotype. Somules were therefore incubated in increasing concentrations of GF109203X, PMA, or U0126, and movies taken after 2, 24 and 48 h and imported into ImageJ to assess the effect of treatments on somule length, area, standard deviation of perimeter (as a proxy for contractile motility), and morphology. In all cases, over 48 h, GF109203X, PMA, and U0126 did not significantly affect the mean overall length or area of somules (data not shown). However, at 2 h the PKC activator PMA (2 μM−20 μM) significantly enhanced somule motility with a maximal ~4-fold increase at 10 μM (p ≤ 0.01; Fig. 6b); no significant effect was seen at 2 h with the PKC inhibitor GF109203X or the MEK/ERK inhibitor U0126 (Fig. 6a,c). Control somules (including those in DMSO vehicle) also appeared to increase motility over time. In contrast, GF109203X (≥2 μM) markedly reduced somule motility after 24 and 48 h, with almost no contractile movement detected at between 5 and 20 μM (p ≤ 0.001; Fig. 6a). On the other hand, U0126 only blunted motility when used at 50 μM (p ≤ 0.001; Fig. 6c).

**Figure 7:**
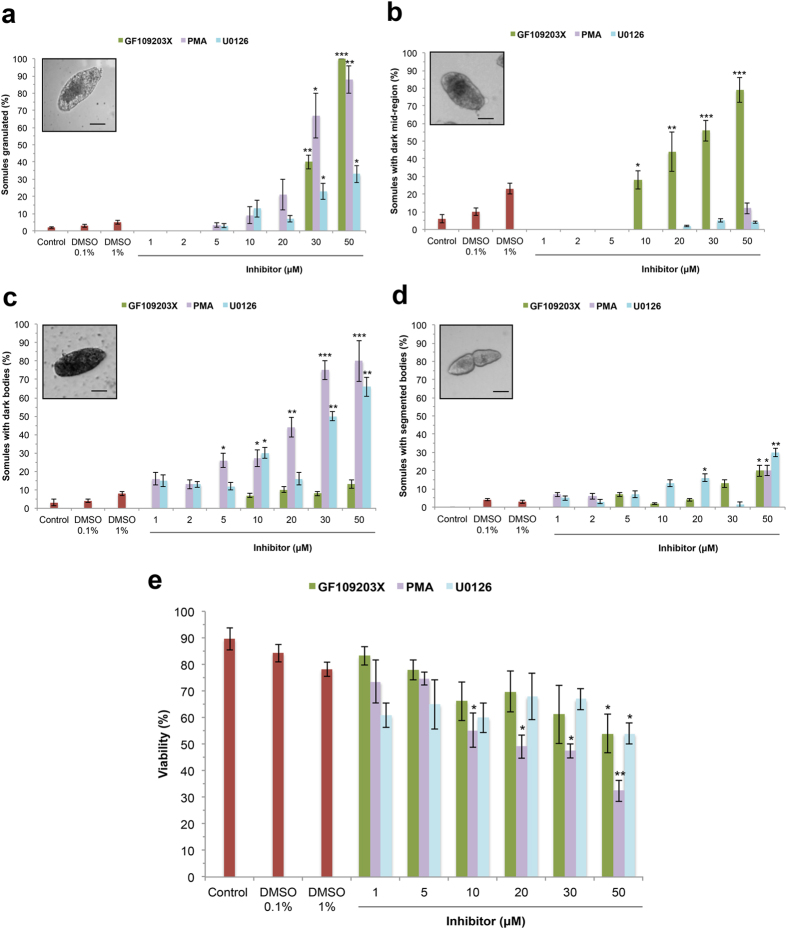
Modulation of PKC or ERK signalling affects morphology and survival of *S. mansoni* somules. (**a–d**) Somules were exposed to increasing concentrations of GF109203X, PMA, or U0126, movies captured at 24 h, and aberrant phenotypes (granulated, dark mid-region, dark body, and segmented body, respectively; shown in insets) enumerated as a percentage of the population observed across three independent experiments (mean ± SEM, n = 30). (**e**) Mean viability ( ± SEM) of somules in response to GF109203X, PMA, or U0126 treatment after 48 h culture. *p ≤ 0.05, **p ≤  0.01, and ***p ≤ 0.001, when compared to respective control values as follows: GF109203X = media (control); PMA and U0126 DMSO: 1–30 μM = DMSO 0.1%, 50 μM = DMSO 1%.

Further visual analysis of movies taken at 24 h revealed that these compounds affected somule morphology. When used at ≥30 μM all three compounds significantly increased the number of somules displaying a degree of granulation (p ≤ 0.05), when compared to their respective controls, with GF109203X having the greatest effect at 50 μM (p ≤ 0.001; Fig. 7a). GF109203X (≥10 μM), in turn caused somules to have darkened mid-region(s) (Fig. 7b), whereas PMA and U0126 induced a more pronounced darkening of the whole somule body (Fig. 7c). Individual somules displaying a segmented body phenotype were also observed, particularly with the highest concentrations of each compound, where over 20% of somules displayed this morphology (Fig. 7d). Finally, with 50 μM PMA the mean proportion of somules displaying swollen bodies (25%) was somewhat greater than DMSO vehicle controls (6%), however the difference did not reach statistical significance (data not shown).

Somule viability was then determined at 48 h using a fluorescence-based assay^31^. Treatment of somules with 50 μM GF109203X resulted in reduced survival from 90% (control) to 54% (p ≤ 0.05) and the effect of U0126 on somule survival was broadly similar to that of GF109203X (Fig. 7e). On the other hand, PMA significantly reduced somule survival when employed at ≥10 μM; death rates of ~60% were observed with 50 μM PMA, when compared to DMSO treated controls (p ≤ 0.01; Fig. 7e).

## Discussion

Focusing on early development of *S. mansoni* somules, we have determined the temporal and spatial activation patterns of PKC and ERK and have characterized their responses to human EGF, insulin, and IGF-I, *in vitro*. The findings highlight the dynamic nature of PKC and ERK signalling in this life stage and demonstrate that human growth factors/hormones have the capacity to modulate schistosome-signalling processes at least *in vitro*; if similar effects occur *in vivo* then it is plausible that such ‘transactivation’ by host molecules could possibly influence the outcome of host infection, schistosome survival and development. Analysis of lipid raft components such as flotillin, and of DRMs, supports the presence of lipid rafts in the parasite tegument, and treatment of somules with MβCD resulted in aberrant ERK activation at the tegument. We therefore hypothesize that lipid rafts in the parasite surface layer are likely to be important to host-parasite communication. Finally, incubation of somules with modulators of PKC and ERK activity revealed that these pathways seem to play a role in somule motility and survival. These protein kinases, that are regulated by host factors, therefore appear to be essential for *S. mansoni* somule homeostasis and may represent suitable drug targets in this and other species of human schistosome.

*In vitro* cultured somules displayed functionally activated PKC and ERK at their surface. Given that the tegument of adult worms displays activated PKC and ERK when fixed immediately after perfusion from mice^15^, we surmise that these kinases would also be activated at the somule surface *in vivo*. Interestingly, PKC/ERK activation also occurred at the somule tegument at 16 h when not exposed to serum. Somules might therefore possess endogenous mechanisms for tegumental PKC/ERK activation or respond to minimal components of BME, which include amino acids. Certain schistosome receptors can be triggered by non-growth factor like ligands, as has been shown in a *Xenopus* expression system with L-arginine, other amino acids, and the RTK-like Venus kinase receptors 1/2 (VKRs 1/2) that possess Venus flytrap (VFT) modules^32^,^33^. Importantly, when used at physiologically relevant concentrations, EGF, insulin and IGF-I activated the 81 kDa PKC and ERK, 116 kDa PKC and ERK, and 116 kDa PKC, respectively and at different times. Because PKC and ERK pathways govern a plethora of biological responses in organisms^11^,^12^,^34^, this finding opens the possibility that host signalling molecules trigger somule development and co-ordinate wide-ranging function in the parasite, with the different ligands influencing different outcomes through differential PKC and ERK signalling, perhaps in a manner that would differ between individual hosts. The EGF-mediated responses are likely delivered through SER, the *S. mansoni* EGF receptor (EGFR) homologue that was found to bind EGF in a *Xenopus* over-expression system^35^, although four putative EGFR proteins have been mined bioinformatically^16^, with two (Smp_152680 and Smp_165470) currently curated in GeneDB. Stimulation of ERK signalling by EGF, which was particularly observed at the tegument, might be important during early host invasion during which the parasite will not only sense human EGF for the first time but must rapidly adapt to the host; thus, host-mediated ERK activation might drive tegument remodelling ensuring parasite survival in addition to promoting cell growth/differentiation. During host infection the somule will, however, be exposed to a complex mixture of host factors and cross talk between pathways will ensue. For example, the transforming growth factor β (TGFβ) pathway, a focus of much research in schistosomes^36^,^37^,^38^,^39^, is well known to crosstalk with the ERK pathway in a non-canonical fashion in humans^40^. Furthermore, in *S. mansoni* the linker region of common mediator (Co-)Smad4 contains three ERK phosphorylation motifs opening the possibility that ERK could restrict interaction of Smad4 with receptor activated (R-)Smad2 to modify TGFβ-mediated outcomes^36^,^41^. Activated protein kinase A (PKA) has also recently been shown to localise to multiple structures in *S. mansoni* somules including the tegument and PKA activation in somules was upregulated by human serotonin and dopamine but was supressed by neuropeptide Y^42^. Perhaps not surprisingly, PKA is known to influence ERK^43^ and PKC^44^ signalling in other systems through cross-talk to allow dynamic and specific control of signalling dependent upon input signal. It would be valuable to explore signal-mediated responses in somules that have penetrated through mouse skin or human skin equivalents to enable us to appreciate how a complex host environment might module multiple signalling events in schistosomes.

With regard to insulin receptors, two types, SmIR-1 and SmIR-2, exist in *S. mansoni* and these can interact with human insulin^45^, with SmIR-1 preferentially localized to the somule tegument together with the glucose transporters STGP1 and SGTP4, and SmIR-2 localized to the parenchyma^45^. In adult worms, the receptor localization was broadly similar with muscular staining also seen in the males^45^. Interestingly, however, despite the similar stimulatory effect of IGF-I on ~116 kDa PKC activation in the current study, this ligand did not interact with either SmIR-1 or SmIR-2 in two-hybrid assays^45^. Thus the putative receptor(s) for the human IGF-I-mediated responses remain elusive even though *S. mansoni* also possess a putative IGF-I (Smp_151640; sourced at GeneDB). In a functional context, human insulin has been shown to increase schistosome glucose uptake whereas RNA interference (RNAi) of the IRs reduces uptake and impacts schistosome development^46^,^47^. Moreover, exposure of *S. japonicum* to human insulin modulated expression of 1,101 genes and, based on the findings of the current research, some of these effects would presumably have been driven through upregulated PKC and ERK signalling. Interestingly, a putative insulin-like peptide has recently been identified in *S. mansoni*^48^, although whether this peptide interacts with the SmIRs remains unknown.

Prior to this research, isolation of DIGs from the tegumental double surface membrane of *S. mansoni* and identification of caveolae-like structures therein suggested that lipid rafts form in this host-interactive layer^25^, providing a hub for certain signalling events. Here, labelling of 24 h somules with CTB after exposure to EGF revealed that GM1 clusters exist at the schistosome surface, further supporting the presence of lipid rafts in *S. mansoni*. Such clusters were also visualized in the tegument of adult worms. Flotillins, evolutionarily conserved proteins that are anchored in cellular membranes including the plasma membrane, are thought to be important to raft organization and are used as DRM and thus lipid raft markers^23^,^49^,^50^,^51^. Using antibodies that target the highly conserved flotillin domain, a flotillin-like protein was found enriched in adult worm DRMs following sub-cellular fractionation and also localized to the tegument, sub-tegument and other regions of 24 h somules. Two other proteins, Ras and G protein (Gq) that are important to ERK and PKC signalling displayed similar *in situ* distribution to the flotillin-like protein, with Ras abundantly associated with the somule surface layer(s). Although we hypothesized that, similar to flotillin, Ras would be enriched in the triton-insoluble DRM fraction it was largely recovered in the triton-soluble fraction. However, not all Ras-related proteins associate with lipid rafts^24^,^52^ likely because of their prenylation/palmitoylation status whereby prenylation can exclude modified proteins from lipid rafts but palmitoylation enhances their raft interaction^20^,^23^,^53^. Furthermore, activation status can affect partitioning of certain Ras isoforms; for example, GTP-mediated activation of H-Ras causes a conformational change that drives it out of lipid rafts^54^. Given that collectively our results further supported the existence of lipid rafts at the surface of schistosomes, we investigated the impact of raft disruption through cholesterol depletion using MβCD on PKC and ERK signalling. Although PKC activation was largely unaffected by MβCD, ERK activation was temporally suppressed by low (1 mM) MβCD concentrations but enhanced with a high (10 mM) dose, particularly at the tegument, supporting a role for cholesterol-rich lipid rafts in transmembrane signalling in schistosomes. It has been reported that EGF stimulation of ERK is enhanced in Rat-1 cells treated with MβCD^55^, but in contrast MβCD treatment alone has been shown to suppress ERK activation in human breast cancer cells^56^. Mechanisms/outcomes of growth factor signalling in rafts are complex and involve, amongst other factors, the architecture of the raft, the affinity of receptors for the raft and the downstream coupling of pathways^21^.

Given that human EGF, insulin and IGF-I stimulated ERK and PKC activities in somules *in vitro* we wished to establish the importance of ERK and PKC activity to somule phenotype. We therefore employed a pharmacological approach^57^ using U0126, GF109203X, or PMA at seven wide-ranging concentrations to affect global PKC or ERK activation in somules. Somule movement was almost completely abolished by low doses (≥2 μM) of GF109203X and a high dose of U0126 (50 μM) at 24 h with effects broadly more potent than those reported in adult *S. mansoni* over a similar duration^15^. Short-term (2 h) incubation with the PKC activator, PMA, stimulated movement whereas longer-term incubation (≥24 h) supressed movement in accord with the down-regulatory effect of this compound on PKC expression after 24 h^15^. Collectively, these findings support roles for PKC and ERK in somule muscular contraction and we hypothesize that PKC/ERK are fundamental to enabling the parasite to migrate within the skin to gain entry to the vasculature, possibly in response to host growth factors. Further visual analysis 24 h after drug treatment revealed that all three compounds (≥30 μM) increased the proportion of granulated somules, with GF109203X (≥10 μM) also enhancing numbers with dark mid-regions and PMA/U0126 (≥10 μM) inducing a more general dark-bodied phenotype; segmented somule bodies were also more prevalent with either compound, particularly when administered at 50 μM. The dose-responsive nature of the effects of these compounds on somule phenotype suggests that GF109203X, U0126, and PMA are acting specifically towards their intended targets in somules. Moreover, these findings emphasise the value of classifying phenotype discretely in such experiments rather than labelling somules as simply ‘normal’ or ‘granulated’. Analysis of somule viability at 48 h using a fluorescence-based assay revealed that not all somules displaying heavily granulated/darkened bodies at 24 h were dead. However, PMA (≥10 μM), and at higher doses GF109203X and U0126 significantly increased somule mortality. Although we did not perform drug ‘wash-out’ experiments to ascertain whether the phenotypic effects were reversible, granulation is often regarded as a phenotype that ultimately results in somule death. While in the present study we aimed to supress global PKC activation, RNAi of a single PKC (Smp_096310; atypical PKCι-type) was recently shown to result in increased somule death after two weeks in the presence of human red blood cells^58^. In a separate study, RNAi of ERK1/2 was performed in *S. mansoni* somules, which were then injected into mice, and although ERK1/2 mRNA levels were supressed, reduced parasite survival *in vivo* was not seen when compared to controls^59^. Nevertheless, egg output by resultant adults decreased 44%, which was in accord with reduced ovary size^59^. In comparison, the increased somule death following ERK inhibition by U0126 observed after only 48 h in the present study could be due to U0126 attenuating ERK pathway activation more potently that was achieved by RNAi (during which ERK1/2 transcripts were supressed by between 92 and 33% on days 2, 4 and 7)^59^.

In summary, this research provides the first insights into host-mediated modulation of ERK and PKC pathways in schistosomes *in vitro* and highlights the importance of these pathways to somule homeostasis. Moreover, further support is provided for the existence of lipid rafts at the schistosome surface and we hypothesize that such rafts mediate the transfer of multiple molecular signals from the host to the parasite that regulate parasite behaviour, growth and development.

## Methods

### Parasites

*Biomphalaria glabrata* snails infected with *S. mansoni* (Strain: NMRI) were provided by the NIAID Schistosomiasis Resource Center of the Biomedical Research Institute (Rockville, MD, USA) through NIH-NIAID Contract HHSN272201000005l distributed through BEI Resources. When patent, snails were placed in filtered tap water (Brimak filter, Silverline) and were exposed to light to induce cercarial emergence; cercariae were then transformed mechanically to somules using an adaptation of published methods^60^,^61^,^62^,^63^. Collected cercariae in Falcon tubes were placed on ice for 15 min, centrifuged (100 *g* for 5 min) and the supernatant discarded; BME containing antibiotics/antimycotics (Sigma) was added to ~4 ml and tubes gently mixed and placed at 37 °C to encourage cercarial movement. Cercariae were next vortexed for 5 min and Hanks Basal Salt Solution (HBSS) added to 7 ml after which tubes were placed on ice for 7 min and re-centrifuged for 2 min to separate the detached tails from heads; this process was then repeated. The supernatant was removed, BME added, and the suspension swirled in a high-walled glass Petri dish to concentrate somules into the centre. The somules were collected, enumerated, loaded into individual wells of 24-well culture plates (Nunc; ~1000 somules/1 ml of BME containing antibiotics/antimycotics), and incubated in 5% CO_2_ at 37 °C. After 16 h somules were transferred into Basch’s medium^64^ and incubated in 5% CO_2_ at 37 °C.

Adult worms were supplied by Bioglab Ltd, c/o Professor Mike Doenhoff, University of Nottingham, UK.

### Evaluating kinase activation during early somule development and in response to human factors

The phosphorylation (activation) status of PKCs and ERKs was determined at 3, 16, 24, 48, 72, and 96 h by western blotting. At each time point somules were transferred to cooled microfuge tubes, placed on ice for 1 min, and pulse centrifuged. Radio immunoprecipitation assay (RIPA) buffer (30 μl) (Cell Signalling Technology (CST), New England Biolabs)/HALT protease/phosphatase inhibitor cocktail (1 μl) (Pierce, Thermo Scientific) was added on ice to lyse the pelleted somules and, after brief (10 s) sonication, 2 μl aliquots were removed for protein quantification using Bradford reagent (Sigma) with bovine serum albumin (BSA) as the protein standard. An appropriate volume of SDS-PAGE sample buffer was added to the remaining lysate and samples heated for 5 min at 90 °C and either electrophoresed immediately or stored at −20 °C. SDS-PAGE performed with 10% Precise pre-cast gels (Pierce) and western blotting with anti-phospho PKC (ζ Thr410), anti-phospho PKC (βII Ser660)), anti-phospho p44/42 MAPK (ERK1/2) (Thr202/Tyr204) antibodies (CST; each at 1/1000) was carried out according to our previously published methods^13^,^15^,^65^,^66^,^67^. Anti-actin antibodies (Sigma; 1:3000) were used to assess protein-loading differences. This was important because of difficulties experienced in obtaining equal numbers of parasites in each sample; phosphorylation levels were then normalised against differences in actin signal between samples^15^,^65^,^68^.

To determine whether PKC or ERK could be activated by host growth factors/hormones, 3-day old somules that had been cultured in Basch’s medium and then starved by washing and culturing in serum-free BME overnight (~16 h) were treated with EGF (Merck; 15 ng/ml), IGF-I (Sigma, 15 ng/ml), insulin (Sigma; 1 μM), or were left untreated (BME, control). Exposure times and concentrations used were adapted from gene expression studies conducted in *Schistosoma japonicum*^69^, and also from work published with EGF in *Trypanosoma brucei*^70^. After exposure (5, 15, 30, and 60 min for EGF/IGF-I; 30 and 60 min for insulin), somules were placed on ice and processed for western blotting as detailed above. In some experiments somules were also maintained in Basch’s medium (and were not washed/further treated) to evaluate the effect of this growth factor-rich medium on protein kinase activation.

*In situ* mapping of functionally activated PKCs and ERKs in somules with anti-phospho PKC (ζ Thr410), anti-phospho PKC (βII Ser660)), anti-phospho p44/42 MAPK (ERK1/2) (Thr202/Tyr204) antibodies (CST; each at 1/50 in 1% BSA in PBS), either during early development *in vitro* or in response to the various growth factor/hormone treatments, was performed according to our published methods^15^,^65^,^66^,^67^. For immunohistochemistry, actin was also stained with anti-actin cy3 conjugated antibodies. Somules were visualised using a Leica SP2 AOBS laser scanning confocal microscope (40x or 63x objectives) and images captured; photomultiplier tube voltages and laser settings were equal for each comparative experiment. Because kinase activation within individual somules of a population might vary slightly, only somules that displayed activation patterns common to the vast majority of those present were selected for image capture.

### Identification of lipid rafts/raft markers

Lipid raft staining in somules and adult worms was done using the AlexaFluor 594 lipid raft labelling kit (Invitrogen). Somules, cultured for 24 h in serum-free BME, were exposed to EGF (15 ng/ml) for 5 min or left untreated and were then placed on ice. Adult worms were also exposed to EGF (15 ng/ml). Parasites were then immediately washed in 2 ml chilled BME and incubated with fluorescent cholera toxin B subunit (CT-B) conjugate (1 μg/ml) for 10 min. Next, parasites were washed thrice with 2 ml PBS before incubating in anti-CT-B antibody solution for 15 min to crosslink the CT-B conjugate. After further PBS washes parasites were pelleted (somules) at 200 *g* in a centrifuge, or (adults) carefully removed, and fixed in ice-cold acetone for 30 min. All of the above incubations and washes were performed on ice. Parasites were then transferred to slides, mounted in Vectashield mounting medium (VectorLabs) and visualized on a Leica SP2 AOBS laser scanning confocal microscope.

To identify commercially available antibodies that might react with *S. mansoni* flotillin, Gq and Ras, protein sequences for *Homo sapiens* flotillin-1 (NP_005794.1), Gq/α11 (NP_002058), and K-Ras (NP_203524) were initially retrieved from the National Center for Biotechnology Information (NCBI: http://www.ncbi.nlm.nih.gov/protein), a BLAST (Basic Local Alignment Search Tool) search performed against *S. mansoni* protein sequences held within GeneDB (www.GeneDB.org), and pair-wise protein alignments constructed using Uniprot Align (http://www.uniprot.org/align/). Antibodies were then selected according to homology within the antibody binding regions. Identified antibodies (anti-flotillin 1 (1/500), 610820, BD Biosciences; anti-Gq/α11 (1/1000), CT|06–709, Millipore; anti-Ras (1/1000), 3965 – CST) were next screened for immunoreactivity against *S. mansoni* somule protein extracts by western blotting as detailed above. Adult worm protein extracts were prepared by homogenizing one worm pair in 30 μl RIPA buffer and processing further as detailed for somules and rat brain lysates were used as a mammalian control. Somules were next processed for immunohistochemistry (as detailed above) with anti-flotillin 1, anti-Gq/α11 and anti-Ras antibodies (each at 1/50) to determine the *in-situ* expression patterns of the respective proteins.

### Preparation of DRMs and disruption of lipid rafts

Preparation of DRMs was achieved according to Adam *et al*.^28^. Adult worms (50 pairs) were homogenized on ice in 150 μl buffer ‘A’ (25 mM 2-(N-morpholino)-ethanesulfonic acid (MES), 150 mM NaCl, pH 6.5, incorporating HALT protease/phosphatase inhibitors) using a motorised microfuge pestle and the detergent-free extracts centrifuged at 500 *g* for 5 min at 4 °C to pellet cellular debris, nuclei and intact cells. The supernatant was then centrifuged at 16,000 *g* for 10 min, 4 °C, and the resultant supernatant retained at the cytosolic fraction. The high–speed pellet was next subject to successive detergent extraction by initially resuspending in buffer ‘A’ and combining with an equal volume of buffer ‘A’ containing 2% triton X-100. Samples were then incubated on ice for 30 min, centrifuged at 16,000 *g* for 20 min at 4 °C and supernatants collected as the triton-soluble (TS) fraction. Pellets were rinsed in buffer ‘A’ and resuspended in buffer ‘B’ (10 mM Tris-HCl, 150 mM NaCl, 60 mM β-octylglucoside, with protease/phosphatase inhibitors), incubated on ice for 30 min, centrifuged at 16,000 *g* for 20 min at 4 °C and supernatants collected as the triton-insoluble (TI) fraction. A 10 μl aliquot of each fraction was removed for protein estimation (using the Bio-Rad detergent-compatible protein assay) and an appropriate amount of 5x SDS-PAGE loading buffer added; samples were then heated and processed for western blotting with anti-flotillin, anti-Ras, and anti-β-tubulin antibodies (1/1000) as detailed earlier.

To assess the effects of raft disruption by cholesterol depletion on signalling, 24 h *in vitro* cultured somules were incubated with MβCD (1 mM or 10 mM) for 5, 10, 20 or 30 min prior to stimulation with EGF (15 ng/ml) for 5 min. Samples were then processed for western blotting with anti-phospho-PKC (ζ Thr410) and anti-phospho p44/42 MAPK (ERK1/2) (Thr202/Tyr204) antibodies as detailed above. Somules obtained at 20 min MβCD/5 min EGF exposure were also processed for immunohistochemistry with anti-phospho p44/42MAPK (Thr202/Tyr204) (ERK1/2) antibodies (above). A single z-scan through the centre of each somule was captured using a Leica SP2 AOBS laser scanning confocal microscope and the fluorescence intensity across the parasite in two randomly selected places was determined using Leica quantification software.

### Functional and viability assays

Newly transformed somules (~300 per well) were maintained overnight (16 h) in individual wells of a 48-well tissue culture plate in 300 μl serum-free BME containing various concentrations (1–50 μM) of GF109203X, U0126, or PMA, or in BME alone/BME plus DMSO (at 0.1% or 1%; vehicle controls for U0126 and PMA). The next day, BME was replaced with Basch medium and pharmacological agents/DMSO added in the same manner. Movies (each 1 min long, 13 frames/s) of somules were recorded with a digital Motic camera attached to a Motic inverted microscope 2, 24 and 48 h after adding the Basch medium (containing components), and the behavioural/morphological effects observed and quantified. Movies were converted to uncompressed AVI using VirtualDub (www.VirtualDub.com) and imported into ImageJ for Windows (www.rsbweb.nih.gov/ij/) running the wrMTrck plugin (www.phage.dk/plugins/download/wrMTrck.pdf). Frames were converted to greyscale, background subtraction and Otsu thresholding applied and somules analyzed with wrMTrck. Three parameters were evaluated: average total somule area, average total somule length, and standard deviation of the perimeter in all observed frames, that latter of which was used as a proxy for determining somule movement (contractions and distensions). When somules were touching each other, results of those individuals were discarded as the software considers them as one object. In addition, a visual analysis of somule phenotype was performed at 24 h with somules categorized as normal, granulated, possessing a darkened mid-region or entire body, or being segmented or swollen.

At 48 h, 100 parasites per treatment were removed and their survival determined using a fluorescein diacetate/propidium iodide (FDA/PI) viability assay^31^. Briefly, somules were transferred to microfuge tubes, centrifuged (200 *g* for 10 s) and each pellet washed twice in warmed (37 °C) PBS before adding 200 μl PBS containing 2 μg/ml PI and 0.5 μg/ml FDA. Immediately after, parasites were transferred to black-walled microtitre plates and fluorescence measured in dual mode with 544 nm excitation/620 nm emission (to detect PI/dead) and 485 nm excitation/520 nm emission (to detect FDA/live) with a FluorStar Optima plate reader. All fluorescence values were obtained at 10 min with the plate reader incubator set at 37 °C to keep temperature constant to ensure sufficient esterase conversion of fluorescein diacetate to fluorescein within the live parasites. Two controls were employed, a positive control comprising untreated somules (live) and a negative control of heat-killed somules (65 °C for 10 min); a blank of PBS/FDA/PI was also used to compensate for any inter-plate variation. In order to ascertain percentage of live and dead somules in each treatment group the following formula was used: viability = live (FDA fluorescence)/(dead (PI fluorescence) * live (FDA fluorescence) × 100, where live (FDA fluorescence) = (sample − negative control)/(pos. control − negative control) and dead (PI fluorescence) = (sample − blank)/(negative control − blank)^31^.

### Statistical analysis

Statistical comparisons were performed with one-way analysis of variance (ANOVA) using Minitab (version 16). All data were expressed as mean ± SEM, and statistical significance was determined by Fisher’s multiple pair-wise comparison.

## Additional Information

**How to cite this article**: Ressurreição, M. *et al*. Molecular characterization of host-parasite cell signalling in *Schistosoma mansoni* during early development. *Sci. Rep.* **6**, 35614; doi: 10.1038/srep35614 (2016).

## Supplementary Material

Supplementary Information
