# Supplementary material for: Molecular characterization of host-parasite cell signalling in Schistosoma mansoni during early development
Source: Sci Rep. 2016 Oct 20;6:35614. doi: 10.1038/srep35614 (PMC5071895; doi:10.1038/srep35614)
Supplement: Supplementary Information [file srep35614-s1.pdf]

Supplementary File

Molecular characterization of host-parasite cell signalling in *Schistosoma mansoni* during early development

Margarida Ressurreição[1], Firat Elbeyioglu[1], Ruth S. Kirk[1], David Rollinson[2], Aidan M. Emery[2], Nigel M. Page[1], and Anthony J. Walker[1*]

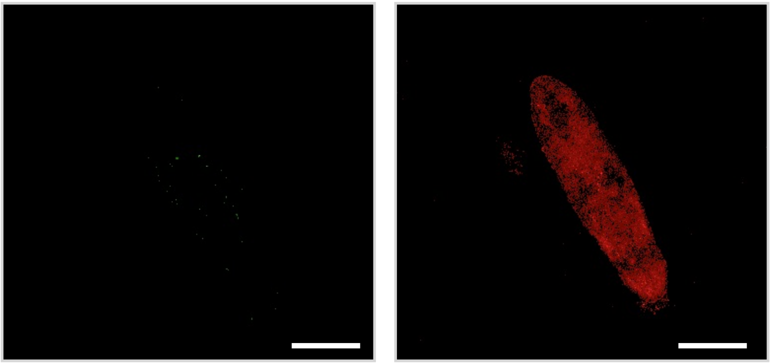

Supplementary Figure 1. Representative confocal micrograph of negative control *S. mansoni* somule. Somules were incubated without primary antibodies but with Alexa Fluor 488 (green) secondary antibodies. Rhodamine phalloidin was used to stain F-actin (red). The background signal from the Alexa Fluor 488/autofluorescence is almost undetectable (left panel). Bar = 25 μm.
